# Number of children and dementia risk: a causal mediation analysis using data from the HUNT study linked with national registries in Norway

**DOI:** 10.1186/s12883-025-04044-4

**Published:** 2025-01-27

**Authors:** Teferi Mekonnen, Vegard Skirbekk, Ekaterina Zotcheva, Bo Engdahl, Bernt Bratsberg, Astanand Jugessur, Catherine Bowen, Geir Selbæk, Hans-Peter Kohler, Jennifer R. Harris, Sarah E. Tom, Steinar Krokstad, Trine Holt Edwin, Yehani Wedatilake, Katrin Wolfova, Dana Kristjansson, Yaakov Stern, Asta Kristine Håberg, Bjørn Heine Strand

**Affiliations:** 1 Department for Physical Health and Aging, Norwegian Institute of Public Health, Oslo, Norway; 2 Norwegian National Centre of Ageing and Health, Vestfold Hospital Trust, Tønsberg, Norway; 3 Centre for Fertility and Health, Norwegian Institute of Public Health, Oslo, Norway; 4 Ragnar Frisch Center for Economic Research, Oslo, Norway; 5 Department of Geriatric Medicine, Oslo University Hospital, Oslo, Norway; 6 Independent Researcher, Vienna, Austria; 7 Department of Global Public Health and Primary Care, University of Bergen, Bergen, Norway; 8 Faculty of Medicine, University of Oslo, Oslo, Norway; 9 Population Aging Research Center and Department of Sociology, University of Pennsylvania, Philadelphia, PA USA; 10 Department of Neurology, Columbia University, Vagelos College of Physicians and Surgeons, New York, USA; 11 Department of Epidemiology, Mailman School of Public Health, Columbia University, New York, USA; 12 Department of Public Health and Nursing, Faculty of Medicine and Health Sciences, HUNT Research Centre, Norwegian University of Science and Technology, Trondheim, Norway; 13 Levanger Hospital, Nord-Trøndelag Hospital Trust, Levanger, Norway; 14 Department of Genetics and Bioinformatics, Norwegian Institute of Public Health, Oslo, Norway; 15 Department of Neuromedicine and Movement Science, Faculty of Medicine and Health Sciences, Norwegian University of Science and Technology, Trondheim, Norway; 16 Department of Epidemiology, Second Faculty of Medicine, Charles University, Prague, Czech Republic

**Keywords:** Dementia, Causal mediation analysis, Number of childern

## Abstract

**Background:**

Childlessness, as well as having a high number of children, has been reported to be associated with an elevated risk of dementia compared to having 2–3 children. The mechanisms underlying these relationships are not well understood and may be mediated by different midlife risk factors. We examined the mediating role of various factors on the relationship between the number of children and dementia risk. These factors include socioeconomic factors (e.g., occupational complexity), psychosocial (e.g.., social activities, loneliness, life satisfaction), lifestyle (e.g., smoking, physical inactivity, alcohol intake), and chronic diseases (e.g., obesity, diabetes, depression, hearing impairment and hypertension).

**Methods:**

Using a historic cohort design, we included 9,745 participants born between 1931–48, with a mean age of 78.2 (SD = 6.4) years at the time of cognitive testing in the HUNT4 70 + sub-study (2017–2019). Further measures were obtained through data linkage between information from Statistics Norway and the HUNT1(1984–86), and HUNT2 (1995–97) Surveys. Causal mediation analyses using an inverse odd weighting approach were conducted to decompose the total effect of the number of children (0, 1, or 4 + children vs. 2–3) on the risk of dementia at age 70 + years into direct and indirect effects with mediators assessed at a mean age of 50.7 (SD = 6.4) years. The analyses were adjusted for age, sex, marital status at age 25 years, educational status, and religion assessed during HUNT3 (2006–2008).

**Results:**

Overall, 15.7% were diagnosed with dementia. The proportions with dementia by the number of children were 22.3% among those with no children, 21.4% for those with one child, 13% for those with 2–3 children (specifically, 12.6% for those with 2 children and 13.4% for those with 3 children), and 19.9% for those with 4 + children. Compared to the reference group of individuals with 2–3 children, the dementia risk was higher among the groups with no children (relative risk (RR): 1.30, 95% confidence interval (CI) (1.12, 1.51)), those with one child (RR: 1.30, 95% CI (1.14, 1.47)) and those with 4 + children (RR: 1.12, 95% CI (1.01, 1.24)). The elevated risks of dementia were not mediated by the socioeconomic, psychosocial, lifestyle, or chronic diseases related factors that we tested. Sex-stratified analysis showed higher dementia risk for men without children and women with one or 4 + children compared to those with 2–3 children, with similar patterns across sexes. None of the mediators contributed to mediation in either group. None of the mediators appeared to contribute through mediation in either group.

**Conclusions:**

Our findings suggest that the number of children—specifically being childless, having one child, or having four or more children—may influence the risk of dementia. These relationships were not mediated by psychosocial, lifestyle, and socioeconomic factors, or markers of chronic diseases in adulthood considered in this study.

**Supplementary Information:**

The online version contains supplementary material available at 10.1186/s12883-025-04044-4.

## Background

Dementia, a condition characterized by symptoms such as memory loss, cognitive deficiencies, and behavioral changes that significantly interferes with a person’s ability to perform daily activities [1]. It is among the most significant public health concerns worldwide, affecting over 55 million people globally [2]. The number of people affected by dementia is estimated to reach 153 million by 2050, in line with the aging global population [3]. Dementia accounts for 11.9% of years lived with disability due to noncommunicable diseases [4] and has adverse effects on national economies [5]. Therefore, public health measures aimed at reducing the incidence/prevalence of dementia are crucial. The 2020 Lancet commission on dementia prevention, intervention, and care [6], highlights that 40% of late-onset dementia cases could be prevented or delayed by targeting modifiable factors. These factors include lower level of education in early life, hearing loss, traumatic brain injury, hypertension, alcohol intake, and obesity in midlife, and smoking, depression, social isolation, physical inactivity, air pollution, and diabetes later in life [6].

Norway is experiencing low fertility rates, with the total fertility rate dropping to 1.4 in 2022 [7–9]. In addition, recent years have seen a shift in parity distributions [10], marked by a trend towards increasing childlessness in newer birth cohorts. For instance, at age 40, about 30% of men and 16% of women in Norway are childless. Given the combination of an aging population and declining fertility rates, it is important to elucidate the specific mechanisms that link the number of children to dementia risk in later-life.

Several studies have identified a U-shaped risk curve, indicating an increased risk of dementia among individuals with 0, 1, or 4 + children compared to those with 2–3 children [11–13].

The specific mechanisms linking the number of children to dementia risk remain unclear; however, factors influenced by whether an individual has no children, too few children, or many children may contribute to the risk of developing dementia in later life among certain groups. For example, childless individuals are often less engaged in social interactions [14, 15], experience loneliness [16, 17], have lower psychological wellbeing [18], and more likely to engage in unfavorable behaviors, such as smoking, alcohol consumption and poor physical activity, [14, 19–22], all of which are associated with increased risk of dementia [6]. On the other hand, having children can promote better social interactions due to child-rearing responsibilities and may encourage favorable lifestyle changes, such as quitting smoking, reducing alcohol consumption [23, 24], achieving greater life satisfaction [25, 26], all of which are protective against dementia [6, 27]. Individuals with many children may also face challenges such as economic strain, reduced working hours, and limited leisure time [28–30], leading to conditions like stress and hypertension that could increase dementia risk. Furthermore, unhealthy lifestyle factors than can be influenced by the number of children an individual has could also increase the risk of chronic diseases (e.g., obesity, diabetes, hypertension, high LDL) [14, 27, 31, 32], which in turn contributes to the risk of dementia later in life [6].

The factors that vary depending on whether an individual has too few or too many children could potentially play a mediating role for the relationship between the number of children and later-life dementia risk. Assessing if, and to what extent, factors influenced by the number of children an individual mediates the relationship between the number of children and dementia risk later in life using formal mediation analysis can help decompose the effect of the number of children on dementia into direct and indirect effects (i.e., through mediating pathways) [33–36]. Understanding how much of the effect is mediated through potential mediating factors is crucial for identifying modifiable risk factors for interventions aimed at reducing overall dementia risk and related differences by number of children. In this regard, prior studies have mainly examined the relationship between the number of children and dementia risk using standard regression techniques [11–13, 37, 38], primarily focusing on direct effects without quantifying the mediating pathways. This approach provides only a partial understanding of the relationship, as it does not reveal the indirect contributions of mediating factors that may be influenced by the number of children an individual has, or how these factors affect their risk of dementia later in life. Studies exploring such mediators in this context are currently lacking.

Additionally, the pathways linking the number of children to dementia risk may vary by sex due to different social roles, responsibilities, and stressors associated with parenthood. For example, among women, increased caregiving responsibilities can make it more challenging to maintain regular physical activity, due to compounded time constraints [19, 20]. They may also be at higher risk of hypertension [39, 40], likely due to cumulative physiological changes from multiple pregnancies and the ongoing stress of raising a large family. The risk of type 2 diabetes is also elevated, especially for those having gestational diabetes [41, 42]. Women with more children often face greater demands on their time [43, 44], which can lead to career interruptions or shifts to more flexible jobs to better manage childcare responsibilities. For men, fatherhood can impact health in ways less directly tied to physical demands. Men’s health risks related to fatherhood may stem from the stress of providing for a family and balancing work and family life. Societal expectations often position men as primary providers, and this socioeconomic pressure can affect their mental health and increase risk factors related to dementia, such as hypertension or depression [45]. However, there is a lack of knowledge regarding the mechanisms for the differential effect of number of children an individual has on dementia risk later in life for men and women.

In this study, we are investigating the idea that the number of children a person has—whether they have no children, one child, or many children (4 or more)—could influence their risk of developing dementia in later life. We think this effect might be mediated by various dementia risk factors. These include socioeconomic factors, such as the complexity of one’s occupation which can indicate the level of cognitive stimulation they are exposed to; psychosocial factors such as social activities, feelings of loneliness, and overall life satisfaction; lifestyle factors such as smoking, physical inactivity, and alcohol consumption; and chronic diseases such as obesity, diabetes, depression, hearing impairment, and hypertension. We propose that these factors could be influenced by whether a person is childless or has too many or too few children. In other words, the number of children a person has could indirectly affect their dementia risk by influencing these factors. In addition, we further explored whether there is sex specific mediating pathways for the relationship between number of children and dementia risk later in life. To address the gap in the literature, we have examined the joint mediating role of socioeconomic, psychosocial, lifestyle, and chronic disease risk factors during midlife on the relationship between the number of children and dementia risk in individuals aged 70 years and older. This was achieved through causal mediation analysis, using high-quality data from the Trøndelag Health Study (HUNT), and linked to data from nationwide registries in Norway.

## Methods

### Study population

The current study used a historical cohort design linking data from older adults aged 70 + years who underwent clinical cognitive assessment in the HUNT4 70 + Study in 2017–19 with administrative prospective data from Statistics Norway and with data from earlier HUNT surveys, HUNT1 (1984–86), HUNT2 (1995–97) and HUNT3 (2006–08). HUNT is a large ongoing general population study initiated in 1984 with the latest survey, HUNT4, completed in 2019 [46]. All adult inhabitants residing in the Nord-Trøndelag County of Norway were invited to participate in all four surveys [46, 47]. The average age of the participants was 45.1 (SD = 6.4) years during HUNT1 and 56.2 (SD = 6.4) years during HUNT2.

**Fig. 1:**
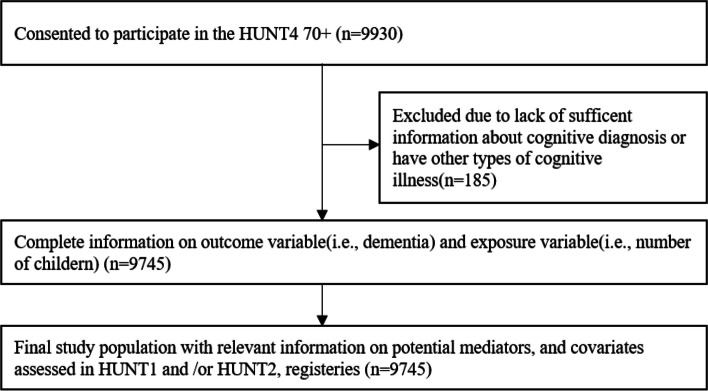
Overview of the sampling scheme: the HUNT Study, Norway

Among the 9930 participants in the HUNT4 70 + study, 185 were excluded due to insufficient information about cognitive diagnosis or the presence of conditions other than mild cognitive impairment or dementia. This resulted in a final study population of 9745 participants born between 1931–1949 (Fig. 1).

### Main exposure variable: number of children

The main exposure variable in our study was the number of children each participant had, categorized into four categories: no children, one child, two to three children, and four or more children. The risk of dementia was similar between the groups who had two vs. three children, and these two categories were thus merged into a single reference group.

### Outcome variable: dementia

The main outcome variable in HUNT4 70 + study was the dementia status of participants, categorized as “yes” vs. “no”. All participants in the study underwent a thorough clinical examination, which included the assessment of cognitive function, daily-life function, neuropsychiatric symptoms, and subjective cognitive decline; interviews with next-of-kin were also conducted. Two medical doctors, from a pool of nine, with expertise in geriatrics, old-age psychiatry, or neurology, used the DSM-5 criteria to classify dementia status. The classifications were as follows: 0) no cognitive impairment, 1) amnestic mild cognitive impairment, 2) non-amnestic mild cognitive impairment, 3) dementia, and 4) either lack of information about cognitive diagnosis or presence of other types of cognitive illnesses [47]. For this study, a dichotomous variable was created: participants in group 3 were classified as “having dementia,” while those in groups 0, 1 and 2 were categorized as “not having dementia”. Group 4 was excluded from the study.

### Potential mediators

We considered potential mediators assessed during HUNT1 and/or HUNT2 to avoid the risk of reverse causality for the mediators assessed during HUNT3. The potential mediators in our study were categorized into four main groups: socioeconomic factors, psychosocial factors, lifestyle factors, and factors related to chronic diseases.

For socioeconomic factors, we considered occupational complexity as a mediator. It was defined into four categories: high complexity, intermediate complexity, low complexity, and not working, based on information from Statistics Norway during HUNT1 and HUNT2 (Table 1).

**Table 1:** Source and definitions of mediating and covariate/confounding variables

Variables	Source	Definition	Confounder and mediator group
**Potential confounders and covariates**			
Age	Statistics Norway	Age in years in 2018	Demographic factors and religion
Sex		Sex was defined as male vs. female	
Educational status		Information on educational status was retrieved from the national registries. It was reported as had no primary or preschool education, completed: primary education, lower secondary education, upper secondary or basic education, upper secondary final year education, post-secondary non tertiary education, undergraduate degree, graduate, and postgraduate study. Then, educational status was defined as completed “secondary school or below” if they completed “final year secondary and post-secondary non-tertiary education or below vs “tertiary education and above” if they completed undergraduate degree or above	
Marital status at age 25 years		In this study population, the mean age at first birth was 25 years and using this information “marital status at age 25 years” was computed. It was defined as married vs. non married/widow/divorce/separated	
Religion	The HUNT study	Religion was retrieved from HUNT3 since this was the only HUNT wave with information on religion. Importantly, we assumed that there is no risk of reverse causation for this variable Religion was defined as being a member of “official religious order” if they responded that they are Christian or other organized religion” vs. identified as “Humanistic or atheistic”	
**Potental mediators**			
Occupational complexity	Statistics Norway	Occupational status information available in 1980(closest available information for HUNT1) and HUNT2 (available in 1995, 1996, and 1997) was used	Socioeconomic factor
		Occupational complexity was defined as ‘‘high complexity’’ if they had professions with international standard classification of occupation (ISCO-88) codes 1–3(e.g., legislators, professionals, technicians), ‘‘intermediate complexity’’ if they had professions with ISCO-884–8 (e.g., clerks, skilled agricultural workers, and machine operators), ‘‘low complexity’’ if they had professions with ISCO-88 code 9 (e.g., cleaners) and ‘‘not working if they had no occupational information/ or being outside the work force	
Participation in social activities	The HUNT study	Participation in social activities (e.g., athletic club) was self-reported in HUNT2. It was defined as participated “never to only few times per year” vs “more than once per week to 1–2 times per month”)	Psychosocial factors
Life satisfaction		Life satisfaction was reported on a Likert scale ranging from 1 (very satisfied) to 7 (very dissatisfied). The coding of the items comprising the life satisfaction scale were reversed such that 1 = very dissatisfied and 7 = very satisfied Then, the average life satisfaction score was computed from HUNT1 and HUNT2 and used on a continuous scale during analysis	
Loneliness		Self-reported loneliness was defined as feeling lonely “very often or sometimes” vs “never or very rarely” during HUNT1 and/or HUNT2	
Smoking		Smoking was defined as “daily smoker” if the participant reported daily smoking at HUNT1, HUNT2 or if they had smoked before and “non-smoker” if they reported not have smoked during HUNT1and/HUNT2 or before	Lifestyle related factors
Physical inactivity		Physical inactivity (yes vs. no) was defined as “yes” if participants did not follow the national recommendations for physical activity (30 min five times per week), assessed based on self-reported frequency, duration, and intensity of weekly physical activity at either HUNT1 and/or HUNT2 and “no” if they did	
Alcohol consumption		Participants’ alcohol consumption was categorized into two groups: “consumed alcohol five times or more” vs “less than five times” during the past two weeks at times of HUNT1 and/or HUNT2	
Obesity		Obesity was defined using body mass index was obtained from HUNT1 and/ or HUNT2, and an individual was defined as having “obesity” if the participants had a body mass index ≥ 30 kg/m^2^ vs “not obese” otherwise	Chronic disease related factors
Hypertension		The participants were defined as having “hypertension” if they have systolic blood pressure of ≥ 140 mm Hg or diastolic blood pressure of ≥ 90 mm Hg and/or used antihypertensive mediations vs not “having hypertension”	
Diabetes		Diabetes (yes vs no) was defined as ‘‘Yes’’ if they had fasting blood sugar level ≥ 7 mmol/liters and/or reporting having diabetes in HUNT1 vs ‘‘No’’ if they had fasting blood sugar < 7 mmol/liters and/or reported they had no diabetes during HUNT1	
Hearing impairment		Hearing impairment (yes vs no) was defined as “yes” if participants reported mild to severe hearing impairment at HUNT1 and/or HUNT2 vs. “no” if they did not	
Depression and anxiety symptoms		Depression and anxiety symptoms were assessed using the Hospital Anxiety Depression Scale (HADS) score completed during the HUNT2 survey and was used as a continuous variable	

Regarding psychosocial factors, information was gathered from self-reported data in HUNT1 and/or HUNT2. These factors included participation in in social activities (dichotomized as never or a few times per year vs. more than once per week to 1–2 times per month), loneliness (dichotomized as feeling lonely very often or sometimes vs. never or very rarely), and life satisfaction (rated on a Likert scale from 1, very dissatisfied, to 7, very satisfied).

Lifestyle factors assessed in HUNT1 and/or HUNT2 included daily smoking (ever vs. never), physical inactivity (yes vs. no), and alcohol consumption (consumed alcohol five times or more times vs. less than five times in the past two weeks).

Chronic disease factors comprised obesity (yes vs. no), hypertension (yes vs. no), diabetes (yes vs. no), hearing impairment (yes vs. no), and depression and anxiety symptoms, which were assessed using a 14-item hospital anxiety depression score. The sources and the definitions of psychosocial factors, lifestyle factors, and chronic disease factors are described in Table 1.

### Potential confounders/covariates

Information regarding the age of participants in 2018, sex (male vs. female), educational status (having completed secondary school or below vs. tertiary education and higher), and marital status at age 25 years (married vs. not married/widowed/divorced/separated) was sourced from Statistics Norway. Religious affiliation (official religious order vs. humanistic or atheistic beliefs) was obtained from HUNT3, was as this was the only wave of the study that included data on religion. Importantly, we assumed that there was no risk of reverse causation for the religion variable. Further details about the sources and definitions of these variables are described in Table 1.

### Statistical analysis

**Fig. 2:**
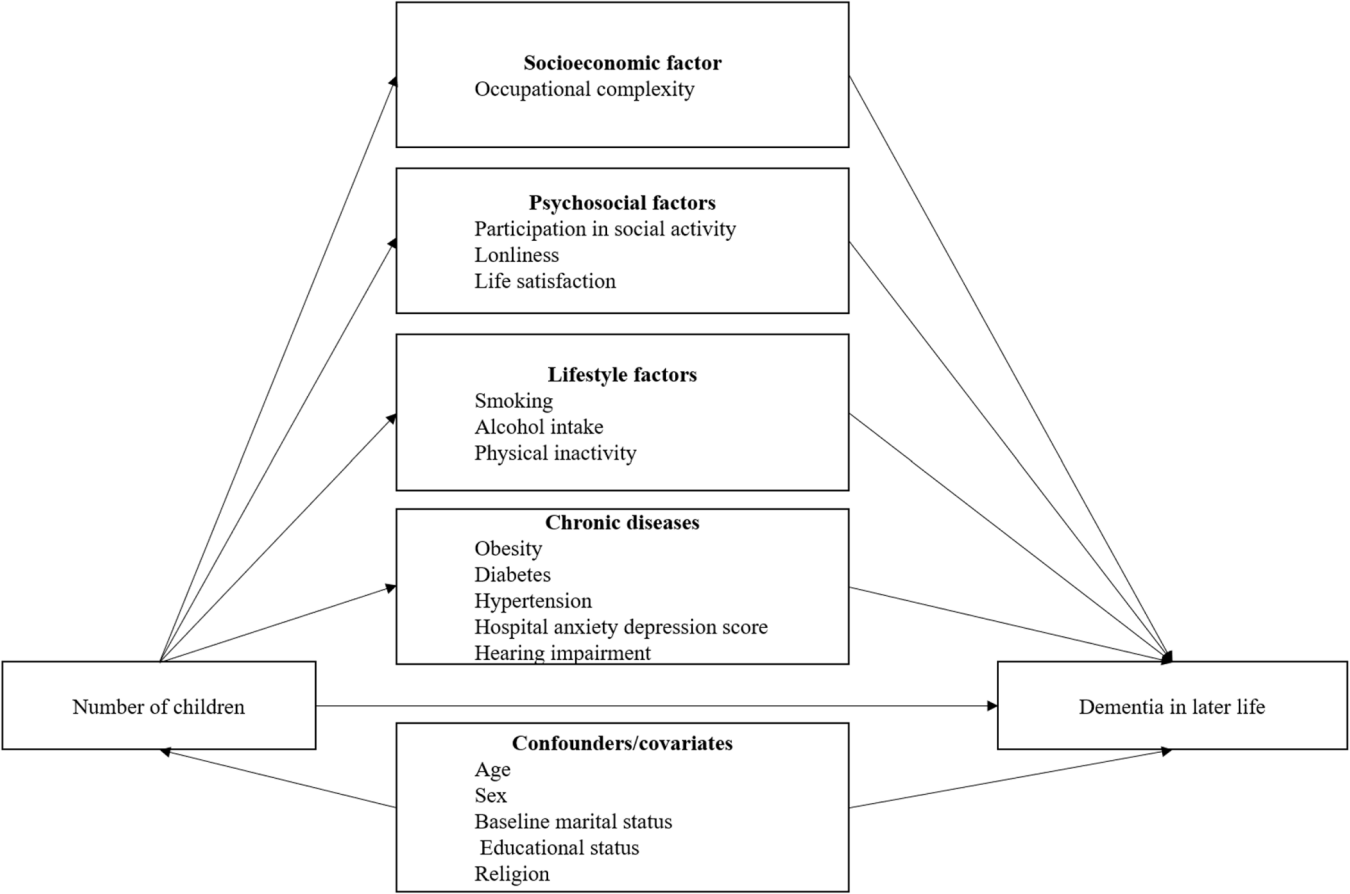
Directed acyclic graph linking number of children to dementia risk

Causal mediation analysis was conducted using an inverse odds weighting approach [36]. We examined the mediating role of various potential mediator groups, as depicted in the direct acyclic graph (DAG) presented in Fig. 2. The analysis focused on decomposing the total effects of having 0, 1, or 4 + children (compared to the reference group of 2–3 children) on dementia risk at age 70 + years into natural direct and natural indirect effects. This decomposition was achieved by using a generalized linear model with Poisson family and the log link function. To estimate the relative risk (RR) of dementia with 95% confidence intervals (CI), a bootstrap method with 200 replications was conducted. We estimated the joint mediating effects of each mediator group individually, as well as all the mediator groups combined. The natural direct effect captures the remaining effect of number of children (0, 1, or 4 + vs. 2–3) on dementia if we were to eliminate the pathway from exposure to the mediators. The natural indirect effect captures the difference between the counterfactual outcomes for an exposed individual (0, 1, or 4 + children) with the mediators set to the value it would normally take when an individual is exposed compared to the same exposed individual with the mediator set to the value it would normally take when the individual is unexposed (2–3 children). The total effect captures how much the outcome would change overall if the exposure status were altered from unexposed (2–3 children) to exposed (0, 1, or 4 + children). Details are found in Supplementary Table 2 and in the causal mediation analyses section of the supplementary file. Missing values were handled using multiple imputations via chained equations using 20 imputed datasets (see Supplementary Table 1). We examined sex-specific mediating pathways for the relationship between number of children dementia risk by running a separate mediation model for men and women. Additional sensitivity analyses were conducted to i) assess the impact of any unmeasured confounder on both mediators and outcome, ii) assess the robustness of the outcome definition, iii) explore if the mediating paths differed for individuals with 0 or 4 + children, and iv) examine if there were education-specific mediating paths for the effect of the number of children on dementia risk (for more details, see the sensitivity analyses section of the supplementary Table 2). In addition, we found no significant interaction effects between sex and the number of children. Hence, sex-specific analyses were not conducted. AGReMA guidelines for observational study was followed for reporting mediation analysis [48]. All analyses were performed using the STATA 16/MP statistical package [49].

## Results

The mean age among the 9745 eligible participants was 70.2 (SD = 6.4) years at HUNT4 70 + (Table 2). Overall, 15.7% had dementia, 6.6% were childless, 7.5% had one child, 33.3% had two children, 32.2% had three children, and 20.5% had 4 + children. The proportion with dementia by number of children was 22.3% among those who were childless, 21.4% among those with one child, 13.0% among those with two–three children, and 19.9% among those with 4 + children. Social activity levels varied by the number of children. Specifically, participants with 0 and 1 child were less socially active, while those with 4 + children were more socially active than those with 2–3 children. Participants with 0–1 children reported more loneliness than those with 2–3 children. Participants with 0, 1, or 4 + children more often reported an unfavorable health lifestyle and a history of chronic diseases compared to those with 2–3 children. Those with dementia were less satisfied in life, less socially active, experienced more loneliness, demonstrated an unfavorable health lifestyle and had a history of chronic diseases outcomes compared to those without dementia (Table 3).

**Table 2:** Characteristics of study participants by number of children (*N* = 9745): The HUNT Study, Norway

	**Number of children**									
	**0**		**1**		**2**		**3**		**4 + **	
**Characteristics**	*n*	*%*	*N*	*%*	*n*	*%*	*n*	*%*	*n*	*%*
Dementia										
* No*	503	77.7	571	78.7	2835	87.4	2,714	86.6	1597	80.1
* Yes*	144	22.3	155	21.3	408	12.6	421	13.4	397	19.9
Sex										
* Women*	294	45.4	382	52.6	1,693	52.2	1,720	54.9	1,211	60.7
* Men*	353	54.6	344	47.4	1550	47.8	1415	45.1	783	39.3
Age in years in 2018 (mean, SD)	79.2	(7.2)	78.7	(7.3)	77.0	(6.1)	77.7	(6.0)	80.3	(6.4)
Education										
* Completed tertiary*	131	20.2	139	19.1	755	23.3	700	22.3	278	13.9
* Completed secondary or below*	516	79.8	587	80.9	2488	76.7	2435	77.7	1716	86.1
Marital status at age 25 years										
* Married*	64	14.0	238	46.2	1795	67.3	1889	74.0	1090	80.4
* Non married/widow/divorced/separated*	394	86.0	277	53.8	872	32.7	663	26.0	265	19.6
Occupational complexity										
* Not working*	15	2.3	27	3.7	119	3.7	117	3.7	93	4.7
* Low*	68	10.5	66	9.1	196	6.0	236	7.5	209	10.5
* Intermediate*	390	60.3	452	62.3	1930	59.5	1914	61.1	1333	66.9
* High*	174	26.9	181	24.9	998	30.8	868	27.7	359	18.0
Religious affiliation										
* Humanistic/atheistic*	62	14.3	88	17.4	406	16.2	321	13.0	150	10.1
* Christian/other*	373	85.7	417	82.6	2099	83.8	2152	87.0	1341	89.9
Hearing impairment										
* No*	555	85.8	611	84.2	2818	86.9	2696	86.0	1681	84.3
* Yes*	92	14.2	115	15.8	425	13.1	439	14.0	313	15.7
Life satisfaction (mean, SD)	5.5	(1.0)	5.7	(1.0)	5.7	(1.0)	5.7	(1.0)	5.6	(1.0)
Participation in social activities										
* Never or only few times/year*	217	49.0	258	47.5	1,021	40.2	920	36.3	593	37.9
* 1–2 times/month to more than once/week*	226	51.0	285	52.5	1,520	59.8	1,611	63.7	971	62.1
Feeling lonely										
* Never/very rarely*	474	89.8	570	90.8	2753	94.0	2762	95.4	1701	93.4
* Sometimes to very often*	54	10.2	58	9.2	175	6.0	132	4.6	121	6.6
Daily smoking										
* No*	282	52.7	268	42.1	1246	41.7	1297	44.3	846	45.6
* Yes*	253	47.3	369	57.9	1739	58.3	1631	55.7	1008	54.4
Physically inactive										
* No*	290	57.1	306	51.4	1620	56.3	1544	54.5	815	47.1
* Yes*	218	42.9	289	48.6	1260	43.8	1289	45.5	914	52.9
Alcohol intake 5 times or more in the past two weeks										
* No*	475	94.1	580	93.7	2631	90.4	2604	91.4	1649	92.8
* Yes*	30	5.9	39	6.3	280	9.6	244	8.6	127	7.2
HADS (mean, SD)	8.3	(5.6)	8.2	(5.8)	7.9	(5.6)	8.0	(5.5)	8.1	(5.6)
Diabetes										
* No*	527	96.5	637	97.1	2987	98.3	2915	97.8	1844	97.7
* Yes*	19	3.5	19	2.9	52	1.7	65	2.2	44	2.3
Hypertension										
* No*	210	38.5	278	42.4	1505	49.6	1427	47.9	787	41.8
* Yes*	336	61.5	378	57.6	1531	50.4	1553	52.1	1098	58.2
Obesity										
* No*	436	80.0	554	84.6	2622	86.4	2472	83.0	1511	80.2
* Yes*	109	20.0	101	15.4	412	13.6	505	17.0	372	19.8

*HADS* Hospital anxiety depression scale

**Table 3:** Characteristics of study participants (*N* = 9745) given as mean, SD, frequencies (%) by dementia diagnosis or not

	Dementia			
	No		Yes	
	n	%	n	%
Total	8220	84.4	1525	15.7
Life satisfaction (mean, SD)	5.7	(1.0)	5.6	(1.1)
Age in years in 2018 (mean, SD)	77.1	(5.7)	83.8	(7.4)
HADS (mean, SD)	7.9	(5.5)	8.6	(5.9)
Number of children				
* 0*	503	6.1	144	9.4
* 1*	571	6.9	155	10.2
* 2*	2835	34.5	408	26.8
* 3*	2714	33.0	421	27.6
* 4* +	1597	19.4	397	26.0
Sex				
* Women*	4400	53.5	900	59.0
* Men*	3820	46.5	625	41.0
Education				
* Completed tertiary*	1843	22.4	160	10.5
* Completed secondary or below*	6377	77.6	1365	89.5
Marital status at age 25 years				
* Married*	4612	67.4	464	65.6
* Non married/widow/divorced/separated*	2228	32.6	243	34.4
Daily smoking				
* No*	3308	43.8	631	45.4
* Yes*	4240	56.2	760	54.6
Physically inactive				
* No*	3128	42.2	514	37.9
* Yes*	4283	57.8	841	62.1
Alcohol intake 5 times or more in the past two weeks				
* No*	6732	91.6	1207	92.2
* Yes*	618	8.4	102	7.8
Participation in social activities				
* Never or only few times/year*	3982	61.4	631	55.3
* 1–2 times/month to more than once/week*	2499	38.6	510	44.7
Feeling lonely				
* Never/very rarely*	6999	94.1	1,261	92.4
* Sometimes to very often*	437	5.9	103	7.6
Occupational complexity				
* Not working*	559	6.8	216	14.2
* Low*	281	3.4	90	5.9
* Intermediate*	5051	61.4	968	63.5
* High*	2329	28.3	251	16.5
Diabetes				
* No*	7541	98.2	1369	96.0
* Yes*	142	1.8	57	4.0
Obesity				
* No*	6473	84.4	1,122	79.0
* Yes*	1200	15.6	299	21.0
Hypertension				
* No*	3740	48.7	467	32.8
* Yes*	3938	51.3	958	67.2
Religion				
* Humanistic/atheistic*	950	14.8	77	7.9
* Member of official religious order*	5489	85.2	893	92.1
Hearing impairment				
* No*	7195	87.5	1166	76.5
* Yes*	1025	12.5	359	23.5

*HADS* hospital anxiety depression scale

### Mediation analyses results

**Fig. 3:**
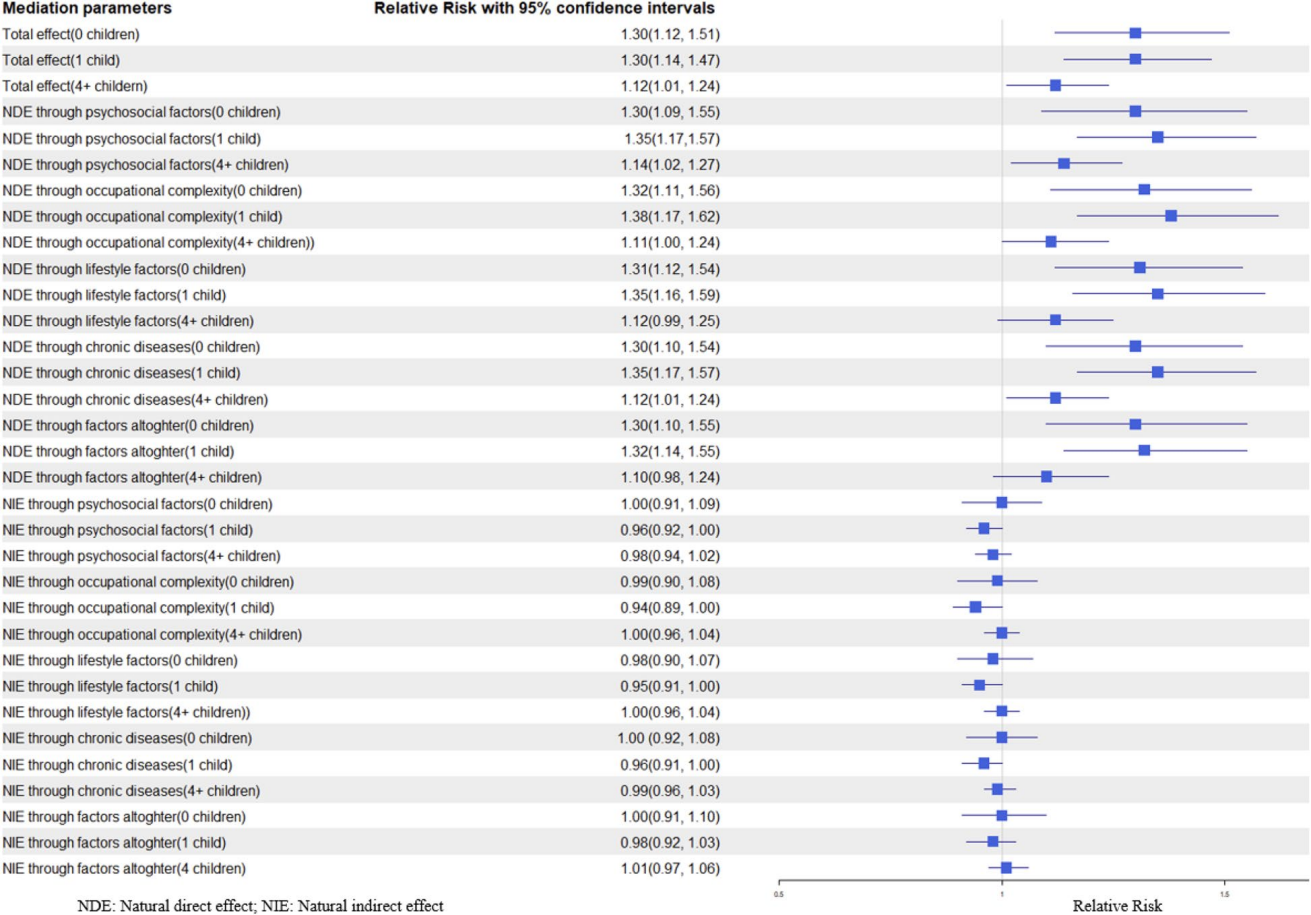
Total effect, natural direct effect, and natural indirect effect of number of children on later-life dementia

Compared to participants with 2–3 children, those who were childless had a higher dementia risk (total effect, RR^TE^: 1.30, 95% CI (1.12, 1.51)), as did those with one child (RR^TE^: 1.30, 95% CI (1.14, 1.47)) and those with 4 + children (RR^TE^: 1.12, 95% CI (1.01, 1.24)) (Fig. 3).

Our analysis indicated that psychosocial factors did not mediate the increased dementia risk associated with having no children (natural indirect effect, RR^NIE^: 1.00, 95% CI (0.91, 1.09)), having one child (RR^NIE^: 0.96, 95% CI (0.92, 1.00)), or having 4 + children (RR^NIE^: 0.99, 95% CI (0.94, 1.02). The direct effect of having 0, 1, and 4 + children on dementia, after accounting for psychosocial factors, was (RR^NDE^: 1.30, 95% CI (1.09, 1.55), 1.35, 95% CI (1.17, 1.57), and 1.14, 95% CI (1.02, 1.27), respectively) (Fig. 3).

Similarly, occupational complexity did not mediate the increased dementia risk among participants who had no children (RR^NIE^: 0.99, 95% CI (0.90, 1.08)), had one child (RR^NIE^: 0.94, 95% CI (0.89, 1.00)), or had 4 + children (RR^NIE^: 1.00, 95% CI (0.96, 1.04)). The direct effect of having 0, 1, and 4 + children on dementia, after accounting for occupational complexity, was (RR^NDE^: 1.32 (1.11, 1.56), 1.38, 95% CI (1.17, 1.62), and 1.11, 95% CI (1.00, 1.24), respectively) (Fig. 3).

Moreover, lifestyle factors did not mediate the total effect on dementia risk among those who had no children (RR^NIE^: 0.98, 95% CI (0.90, 1.07)), had one child (RR^NIE^: 0.95, 95% CI (0.91, 1.00)), or had 4 + children (RR^NIE^: 1.00, 95% CI (0.96, 1.04)). The direct effect of having 0, 1, and 4 + children on dementia, after accounting for lifestyle factors, was (RR^NDE^: 1.31, 95% CI (1.12, 1.54), 1.35, 95% CI (1.16, 1.59), and 1.12, 95% CI (0.99, 1.25), respectively) (Fig. 3).

Similarly, exposure to markers of chronic diseases in midlife did not mediate the total effect on dementia risk for those who were childless (RR^NIE^: 1.00, 95% CI (0.92, 1.08)), had one child (RR^NIE^: 0.96, 95% CI (0.91, 1.00)), or had 4 + children (RR^NIE^: 0.99, 95% CI (0.96, 1.03)). The direct effect of having 0, 1, and 4 + children on dementia, after accounting for a history of chronic diseases during midlife, was (RR^NDE^: 1.30, 95% CI (1.10, 1.54), 1.35, 95% CI (1.17, 1.62), and 1.12, 95% CI (1.01, 1.24), respectively) (Fig. 3).

When evaluating the joint effect of all factors combined, there were no indirect effects on dementia risk among individuals who had no children (RR^NIE^: 1.00, 95% CI (0.91, 1.10)), had one child (RR^NIE^: 0.98, 95% CI (0.92, 1.03)), and had 4 + children (RR^NIE^: 1.01, 95% CI (0.97, 1.07)) compared to those with 2–3 children. The direct effect of having 0, 1, and 4 + children on dementia, after accounting for a history of chronic diseases during midlife, was (RR^NDE^: 1.30, 95% CI (1.10, 1.55), 1.32, 95% CI (1.14, 1.55), and 1.10, 95% CI (0.98, 1.24), respectively) (Fig. 3).

### Mediation analysis results by sex

**Fig. 4:**
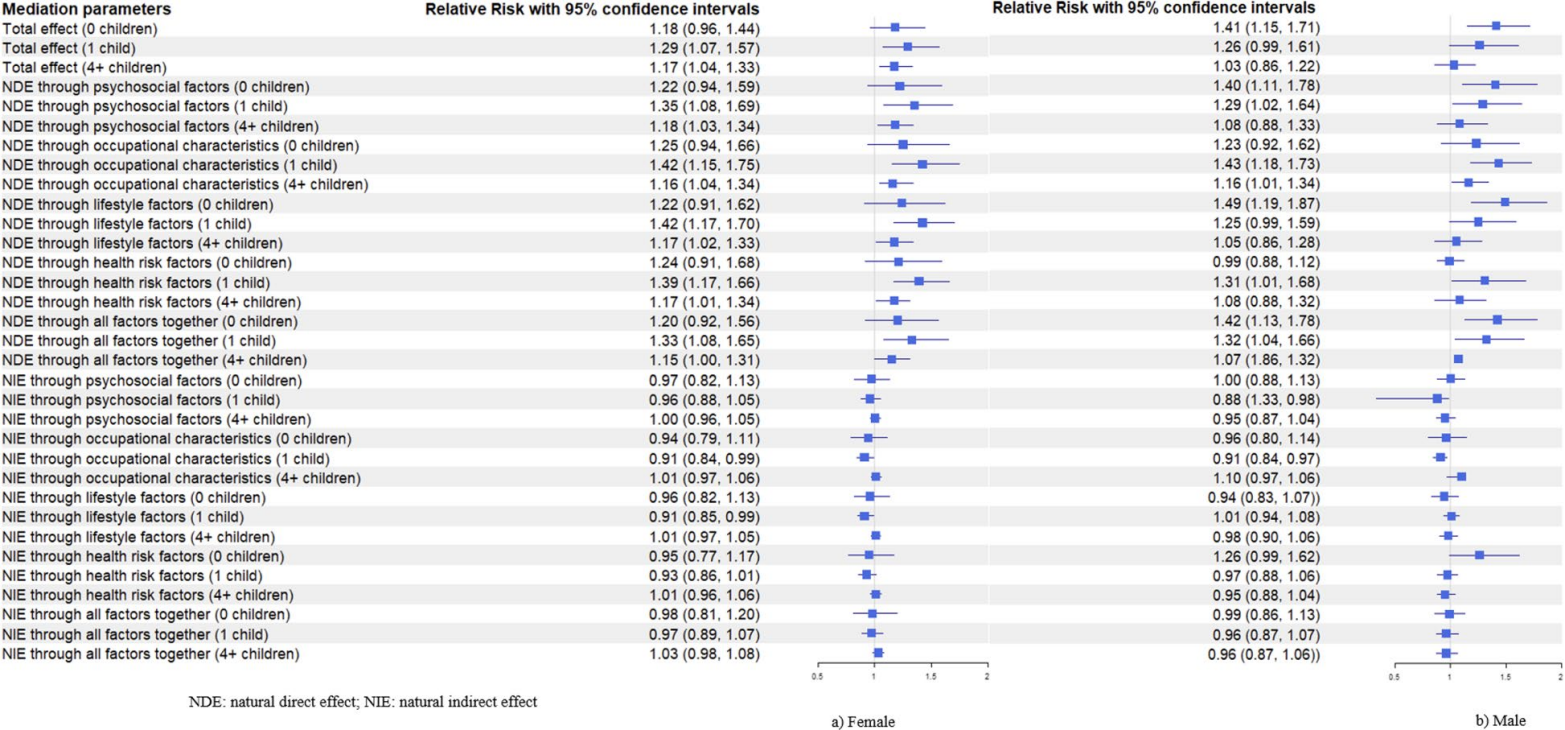
Total effect, natural direct effect, and natural indirect effect of number of children on later-life dementia by sex

Compared to those with 2–3 children, childless men had a higher risk of dementia (total effect, RR^TE^: 1.41, 95% CI: 1.15–1.71), as did men with one child (RR^TE^: 1.26, 95% CI: 0.99–1.61) and men with four or more children (RR^TE^: 1.03, 95% CI: 0.86–1.22) (see Fig. 4). Similarly, in women, childless women showed a higher dementia risk (RR^TE^: 1.18, 95% CI: 0.96–1.44), as did those with one child (RR^TE^: 1.29, 95% CI: 1.07–1.57) and four or more children (RR^TE^: 1.17, 95% CI: 1.04–1.33) (see Fig. 4). None of the mediators demonstrated a mediated effect in either sex.

### Sensitivity analyses results

In our analysis, only E-values for the natural direct effect are presented, as there were no observed indirect effects. The result showed that with an observed natural direct effect (RR^NIE^) of 1.30, 1.32, and 1.10 among those with 0, 1 child and 4 + children, respectively, in the model for all the mediators together, an unmeasured confounder that was associated with number of children and dementia by RRs of 1.92-fold each among childless, 1.97-fold each for those with 1 child and 1.43-fold each among 4 + children, conditional on the measured confounders, could fully explain the natural direct effect estimates, but weaker unmeasured confounder associations could not. The sensitivity analysis excluding those with mild cognitive impairment from the analysis and outcomes defined by including those with mild cognitive impairment to the groups with dementia showed a negligible effect on the mediation parameter estimates (Supplementary Tables 1 and 2). Analyses where data was split into having 0 child vs. 2–3 children and having 4 + children vs. 2–3 children revealed no significant differences in the mediating paths between those without children and those with 4 + children (data not shown). Further sensitivity analyses, in which we divided the data by educational level, also showed no significant differences in the mediation parameters regarding the effect of the number of children on late-onset dementia risk (Supplementary Table 3).

## Discussion

In this large Norwegian population-based historic cohort study, individuals with 0, 1 or 4 + children were found to have a higher dementia risk compared to those with 2–3 children. Contrary to our initial hypothesis, this increased risk was not attributable to differences in midlife psychosocial factors, socioeconomic position, lifestyle, or chronic disease markers.

Our observation of a U-shaped association between the number of children and increased dementia risk aligns with findings from other studies exploring this relationship [11–13, 37, 38, 50, 51]. We initially hypothesized that the higher dementia risk associated with having 0,1 and 4 + children may be influenced by the combined effects of various factors in midlife: socioeconomic position (with occupational complexity as a proxy for cognitively stimulating environments), psychosocial factors (such as participation in social activity, loneliness, life satisfaction), lifestyle factors (including smoking, physical inactivity, alcohol intake), and markers of chronic diseases (obesity, diabetes, depression score, hearing impairment, and hypertension). However, our results did not support this hypothesis, as these factors did not mediate the observed increased risks of dementia associated with having 0, 1 or 4 + children. The absence of mediating effects in our study may partly be due to unmeasured and complex multifactorial mediators, which were unavailable for us, such as quality of life, diet, the amount and quality of sleep, and various forms of social support, which potentially vary by the number of children [52–54]. Given that there was a substantial direct effect on dementia risk of having no children or having one child, even after accounting for potential mediators, further research is needed to fully understand the underlying mechanisms.

The pathways through which having 0 or 4 + children influence later life dementia risk might differ. Childless individuals are likely to have smaller social networks and engage in unfavorable lifestyle behaviors, and they may be at a higher risk of chronic diseases outcomes [14, 55]. In contrast, those with 4 + children may face higher economic strain, contributing to adverse health conditions such as stress and hypertension [28–30]. Our separate analyses, comparing the mediating paths for individuals with 0 and 4 + children to those with 2–3 children, showed no differences in the mediation parameters. However, individuals with 0 or 1 child exhibited a 30% higher later life dementia risk compared to those with 2–3 children. Given the existing evidence of Norway’s lower fertility rates compared to some countries globally [7–9] and the rise in childlessness in recent cohorts [56], it is crucial to support individuals in fulfilling their reproductive preferences. Furthermore, providing assistance to those with a substantial number of children could help mitigate the impact of varying fertility rates on the risk of dementia later in life.

Our stratified analysis by sex indicated that the total effect of having 0, 1, or 4 + children on the risk of dementia, compared to those with 2–3 children, was comparable for both men and women with a higher risk among childless men and women with 1 and 4 + children. None of the considered in our study (i.e., socioeconomic status (e.g., occupational complexity), psychosocial aspects (e.g., social activities, loneliness, life satisfaction), lifestyle choices (e.g., smoking, physical inactivity, alcohol intake), and markers of chronic diseases (e.g., obesity, diabetes, depression, hearing impairment, and hypertension) did not show mediated effects in either sex. Further research is warranted to explore the potential underlying mechanisms.

There is some evidence to suggest that cognitive health and behavior, including educational attainment and socioeconomic status, both of which are related to dementia risk, might affect family planning decisions [57, 58]. Many studies cite length of education as a reason for why women with more education and higher socioeconomic status tend to have fewer children [58, 59]. Studies have shown that individuals with higher cognitive reserve, often reflected in higher educational attainment and socioeconomic status, tend to have different fertility patterns compared to those with lower cognitive reserve. One study found that in both men and women, those with two or three offspring had significantly better cognitive function compared to those without offspring [60]. Another study found that having children was associated with better cognition for men, but not for women [61]. However, these factors were only associated with cognitive functioning, as the individuals were not diagnosed with ADRD. Such studies would require much longer follow ups to fully understand the long-term impacts on ADRD. Additionally, underlying genetic predispositions to dementia could theoretically influence neurological development and subsequent life choices, including the number of children. However, we are not aware of any direct causal evidence linking dementia liability specifically to fertility decisions and this requires further study. Such studies would face several practical challenges. Although educational attainment and socioeconomic status are both associated with ADRD risk, the relationship between these factors and parity may not be directly influenced by the knowledge of one’s own ADRD risk as ADRD typically occurs much later in life, very few individuals are apt to do genetic testing for ADRD susceptibility unless there are several family members affected, and there are several competing causes of death.

The outcome variable in this study was defined by excluding those either lack of information about cognitive diagnosis or presence of other types of cognitive illnesses [47]. Excluding participants with incomplete outcome information could introduce selection bias, impacting both direct and indirect effects. If the excluded group has distinct characteristics affecting the mediator or outcome, this could bias mediator distribution and direct effects, potentially misrepresenting the true relationship between exposure and outcome. In our analysis, efforts were made by applying weights to adjust for potential nonresponse bias and multiple imputation to ensure that the remaining sample represents the larger population. Although, efforts have been made to account for non-response and missing data, it is difficult to quantify to what extent exclusion of these groups from the analysis influences our mediation parameters. Future research should consider using external data or other resources to perform a comprehensive quantitative bias analysis, addressing bias in complex multiple-mediator models like ours.

Furthermore, mediation analysis requires the identification of assumptions of no unmeasured confounding in the exposure-mediator, mediator-outcome, and exposure-outcome relationships [62–64]. However, the exposure variable (i.e., number of children), the outcome variable (i.e., dementia), and the potential mediators, such as lifestyle and markers of chronic diseases, could be affected by various unmeasured factors. This makes the assumptions of no unmeasured confounding challenging, as many of the residual variables are either inaccessible or unknown. In our analysis, we conducted a sensitivity analysis using mediational E-value to assess the possible influence of unmeasured confounder on direct and indirect effects [65]. The results from mediational E-value analysis indicated that to explain away an observed direct effect, an unmeasured confounder, would have to be relatively strongly associated with both dementia and the number of children.

### Strength and limitations

The strengths of this study include the large sample size, population-based sampling standardized approach to diagnosis of dementia, use of high quality registry data for number of children, application of a life-course approach, reduction of missingness using multiple imputation and application of causal mediation analysis [35].

As with many other observational studies, ours is not without limitations. The mediation analysis method we have employed does not show the independent contributions of each mediator, which precludes the identification of a single mediator that is actionable in an intervention. Mediation analysis requires identification assumptions of no unmeasured confounding [62–64], yet the outcome variable, dementia, and the potential mediators, such as lifestyle and markers of chronic diseases, could be affected by various unmeasured factors. An additional limitation of the inverse odds weighting approach in mediation analysis is that this method does not account for time-varying exposures or mediators. This limitation means that the approach may not fully capture dynamic relationships where exposures or mediators change over time, potentially leading to biased estimates if these time-dependent variations significantly impact the outcome. Future studies could address this by employing models that can accommodate time-varying factors, thereby providing a more nuanced understanding of causal pathways in longitudinal data. Assuming that there is no confounder on the mediator-outcome relationships could be challenging as many of the residual variables are either inaccessible or unknown.

There is healthy selection bias in our study as participation in HUNT surveys depend on survival, socioeconomic status, and absence of chronic diseases [66]. Ignoring competing risk, as we have done, might introduce bias [67]. The indirect effect estimates for the factors such as lifestyle behaviors and chronic diseases might be underestimated [68, 69], if there is competing risk, as reported in smoking [67].

## Conclusions

Our findings suggest that the number of children—specifically being childless, having one child, or having four or more children—may influence the risk of dementia. These relationships were not mediated by psychosocial, lifestyle, and socioeconomic factors, or markers of chronic diseases in adulthood considered in this study.

## Supplementary Information

Supplementary Material 1.

Supplementary Material 2.

## Data Availability

The data used in the current study are available after approval by the Regional Committee for Medical and Health Research Ethics and HUNT's Data Access Committee.
