# Supplementary material for: Number of children and dementia risk: a causal mediation analysis using data from the HUNT study linked with national registries in Norway
Source: BMC Neurol. 2025 Jan 27;25:39. doi: 10.1186/s12883-025-04044-4 (PMC11770967; doi:10.1186/s12883-025-04044-4)
Supplement: Supplementary file 1 — Supplementary Material 1. [file 12883_2025_4044_MOESM1_ESM.docx]

**Supplementary files**

Supplementary Table 1: Percentage of missing on the potential mediators and confounders/covariates(n=9745)

| Variable | Percentage missing (%) |
| --- | --- |
| Participation in social activities | 21.8 |
| Loneliness | 9.7 |
| Life satisfaction | 11.2 |
| Smoking | 8.3 |
| Physical inactivity | 10.1 |
| Alcohol consumption | 11.1 |
| Obesity | 6.7 |
| Diabetes | 6.5 |
| Hypertension | 6.6 |
| Anxiety and depression scores | 14 |
| Religious affiliation | 24 |
| Marital status at age 25 years | 22.6 |

**Causal mediation analysis**

We conducted inverse odds weighting approach to causal mediation analysis[1]. The inverse odds weighting method estimates the joint mediation effect of multiple mediators regardless of their scale and accommodates exposure–mediator or mediator–mediator interactions. The total effects of number of children (0, 1, or 4+ vs. 2-3 (reference group)) on dementia risk at age 70+ years were decomposed (within the potential outcome’s framework) into natural direct and natural indirect effects. This was achieved by using a generalized linear model with Poisson family and the log link function. The lack of accommodating such interactions is the main shortcoming of traditional regression-based mediation analysis approaches [1, 2]. The natural indirect effect captures the remaining effect of number of children (0, 1, or 4+ vs. 2-3) on dementia if we were to eliminate the pathway from exposure to the mediators. The natural indirect effect captures the difference between the counterfactual outcomes for an exposed individual (0, 1, or 4+ children) with the mediators set to the value it would normally take when an individual is exposed compared to the same exposed individual with the mediator set to the value it would normally take when the individual is unexposed (2-3 children). The total effect captures how much the outcome would change overall if the exposure status were altered from unexposed (2-3 children) to exposed (0, 1, or 4+ children). Causal mediation analysis assumes that there is no unmeasured confounding of (i) exposure-outcome, (ii) exposure-mediators, (iii) mediator-outcome relations, and iv) no confounder of mediator-outcome relationships affected by exposure variable given measured confounders [3]. Supplementary Table 2 describes mediation analyses models for the main and sensitivity analyses.

| Supplementary Table 2: mediation analysis models | |
| --- | --- |
| Outcome variable | Dementia (no, yes) |
| Exposure variable | number of children (having 0, 1, 4+ vs. 2-3 children) |
| **Potential mediators** | |
| 1. Socioeconomic factor | occupational complexity (a proxy for cognitively stimulating environments) |
| 1. Psychosocial factors | participation in social activities, loneliness, life satisfaction |
| 1. Lifestyle-related factors | smoking, physical inactivity, alcohol intake |
| 1. Chronic non-communicable diseases | obesity, diabetes, hospital anxiety depression score, hearing impairment and hypertension |
| **Confounders/ covariates** | |
|  | age in years in 2018 |
|  | sex of the participant |
|  | marital status at age 25 years |
|  | participation in religious organization |
|  | educational status |
| **Main mediation analyses models** | |
| Model 1 – mediation model for socioeconomic factor | assessed the mediating role of occupational complexity adjusted for confounders/covariates numbered 1-5 |
| Model 2 - mediation model for psychosocial factor | assessed the mediating role of psychosocial factors adjusted for confounders/covariates numbered 1-5 |
| Model 3 - mediation model for lifestyle-related factors | assessed the mediating role of lifestyle-related factors adjusted for confounders/covariates numbered 1-5 |
| Model 4 - mediation model for chronic non-communicable diseases factors | assessed the mediating role of chronic non-communicable diseases factors adjusted for confounders/covariates numbered 1-5 |
| Model 5 - model for all factors combined | assessed the mediating role of all mediators 1-4 altogether adjusted for confounders/covariates numbered 1-5 |
| **Sensitivity analysis mediation models** | |
| 1. Sensitivity analysis for the outcome definition | Two separate mediation models that includes all mediators numbered 1-4 were run for the outcome (i) including those with mild cognitive impairment and dementia in the same group  (ii) excluding those with mild cognitive impairment from the analysis |
| 1. Sensitivity analysis by number of children | After splitting the data by number of children 0 children vs 2-3 children, and 4+ children vs 2-3 children, two separate mediation models were run. The models were i) the mediating role of psychosocial, lifestyle factors, and chronic diseases, in the relationship between having 0 child vs. 2-3 children and dementia and ii) mediation model that includes lifestyle factors and chronic diseases (specifically, depression and hypertension) for the relationship between having 4+ children vs. 2-3 children and dementia. |
| 1. Sensitivity analysis by level of education | After splitting the data by level of education (completed secondary school or below vs. tertiary education and above), a separate mediation model accounting all factors combined were run for “completed secondary school or below” and “tertiary education and above” |
| 1. Mediational E-value estimation | We conduced sensitivity analyses to assess how strongly any unmeasured confounder would have to be related to both the mediators and the outcome to substantially change the conclusions by using mediational E-value[4] . Mediation E-value for the natural direct effect was estimated using mediational E-value formula, RR+$\surd$ (RR x (RR-1)), since there were no indirect effects. Mediational E-values indicate the minimum strength of association the unmeasured confounders would have to be related exposure-outcome, and mediator-outcome relationships to explain away the natural direct and indirect effects estimates conditional on measured confounders/covariates[4, 5]. |

References

1. Nguyen QC, Osypuk TL, Schmidt NM, Glymour MM, Tchetgen Tchetgen EJ: **Practical guidance for conducting mediation analysis with multiple mediators using inverse odds ratio weighting**. *American journal of epidemiology* 2015, **181**(5):349-356.

2. Tchetgen Tchetgen EJ: **Inverse odds ratio‐weighted estimation for causal mediation analysis**. *Statistics in medicine* 2013, **32**(26):4567-4580.

3. VanderWeele T: **Explanation in causal inference: methods for mediation and interaction**: Oxford University Press; 2015.

4. Smith LH, VanderWeele TJ: **Mediational E-values: approximate sensitivity analysis for unmeasured mediator–outcome confounding**. *Epidemiology (Cambridge, Mass)* 2019, **30**(6):835.

5. VanderWeele TJ, Ding P: **Sensitivity analysis in observational research: introducing the E-value**. *Annals of internal medicine* 2017, **167**(4):268-274.

Supplementary Table 3: Total effect, natural direct effect, and natural indirect effect of number of children on dementia excluding those with MCI: the HUNT study (n = 6340)

|  |  | | |
| --- | --- | --- | --- |
| Mediation parameters | Number of children (reference is having 2-3 children) | | |
|  | 0  RR (95%CI) | 1  RR (95%CI) | 4+  RR (95%CI) |
| Total effect, RR^TE^ | 1.32 (1.18, 1.47) | 1.29 (1.17, 1.41) | 1.14 (1.01,1.29) |
| Natural direct effect, RR^NDE^ | 1.28 (1.13, 1.45) | 1.31 (1.18, 1.45) | 1.12(0.99,1.26) |
| Natural indirect effect, RR^NIE^ | 1.03 (0.94, 1.13) | 0.98 (0.91, 1.06) | 1.02 (0.96, 1.07) |

The model is adjusted for age, sex, baseline marital status, educational status, and religion; exposure variable reference: having have two to three children; Mediators: participation in social activity, loneliness, life satisfaction, occupational status, smoking, physical inactivity, alcohol intake, obesity, diabetes, depression, hypertension, and hearing impairment; MCI: mild cognitive impairment.

Supplementary Table 4: Total effect, natural direct effect, and natural indirect effect of number of children on dementia/MCI vs. no dementia/MCI): the HUNT study (n = 9745)

|  |  | | |
| --- | --- | --- | --- |
| Mediation parameters | Number of children (reference is having 2-3 children) | | |
|  | 0  RR (95%CI) | 1  RR (95%CI) | 4+  RR (95%CI) |
| Total effect, RR^TE^ | 1.14 (1.03, 1.24) | 1.14 (1.1.04, 1.24) | 1.02 (0.96,1.09) |
| Natural direct effect, RR^NDE^ | 1.07 (1.01, 1.16) | 1.11 (1.01, 1.21) | 1.01 (0.94,1.07) |
| Natural indirect effect, RR^NIE^ | 1.05 (1.02, 1.09) | 1.03 (1.00, 1.06) | 1.02 (1.01, 1.03) |

The model is adjusted for age, sex, baseline marital status, educational status, and religion; exposure variable reference: having have two to three children; Mediators: participation in social activity, loneliness, life satisfaction, occupational status, smoking, physical inactivity, alcohol intake, obesity, diabetes, depression, hypertension, and hearing impairment; MCI: mild cognitive impairment.

Supplementary Table 5: Total effect, natural direct effect, and natural indirect effect of number of children on dementia vs. no dementia/MCI splited by level of education: the HUNT study (n = 9745)

|  | Tertiary education and above (n = 2003) | | | Secondary school or below (n = 7742) | | |
| --- | --- | --- | --- | --- | --- | --- |
| Mediation parameters | Number of children (reference is having 2-3 children) | | | Number of children (reference is having 2-3 children) | | |
|  | 0  RR (95%CI) | 1  RR (95%CI) | 4+  RR (95%CI) | 0  RR (95%CI) | 1  RR (95%CI) | 4+  RR (95%CI) |
| Total effect, RR^TE^ | 1.45 (0.95, 2.23) | 0.63 (0.25, 1.57) | 1.33 (1.00,1.77) | 1.24(1.14, 1.35) | 1.36(1.18, 1.46) | 1.10(0.97, 1.24) |
| Natural direct effect, RR^NDE^ | 1.78 (0.86, 3.67) | 0.57 (0.23, 1.39) | 1.40 (0.53,3.70) | 1.19(1.08, 1.32) | 1.41(1.25, 1.57) | 1.06(0.94, 1.20) |
| Natural indirect effect, RR^NIE^ | 0.82 (0.53, 1.26) | 1.09 (0.77, 1.54) | 0.95 (0.42,2.16) | 1.04 (0.96, 1.13) | 0.96 (0.88,1.04) | 1.03(0.97, 1.09) |

The model is adjusted for age, sex, baseline marital status, educational status, and religion; exposure variable reference: having have two to three children; Mediators: participation in social activity, loneliness, life satisfaction, occupational status, smoking, physical inactivity, alcohol intake, obesity, diabetes, depression, hypertension, and hearing impairment; MCI: mild cognitive impairment.
